# Early diagnosis and intervention in congenital lower urinary tract obstruction: time to revise our approach?

**DOI:** 10.1007/s00467-025-06994-w

**Published:** 2025-11-13

**Authors:** Jaap Mulder, Stefan Kohl, Federica Fontanella, Alina C. Hilger, Heiko Reutter, Raimund Stein, Eva C. Weber, Rik Westland

**Affiliations:** 1 Division of Nephrology, Department of Pediatrics, Sophia Children’s Hospital, Erasmus Medical Center, Rotterdam, The Netherlands; 2 Division of Nephrology, Department of Pediatrics, Willem-Alexander Children’s Hospital, Leiden University Medical Center, Leiden, The Netherlands; 3 Department of Pediatrics and Adolescent Medicine, Medical Faculty and University Hospital Cologne, University of Cologne, Cologne, Germany; 4 Department of Obstetrics and Gynecology, University Medical Centre of Groningen, Groningen, The Netherlands; 5 Department of Pediatrics and Adolescent Medicine, University Hospital Erlangen, Erlangen, Germany; 6 Research Center On Rare Kidney Diseases (RECORD), University Hospital Erlangen, Erlangen, Germany; 7 Division of Neonatology and Pediatric Intensive Care, Department of Pediatrics and Adolescent Medicine, University Hospital Erlangen, Erlangen, Germany; 8 Center for Pediatric, Adolescent and Reconstructive Urology, Medical Faculty, University Medical Center Mannheim, Mannheim Heidelberg University, Mannheim, Germany; 9 Division of Prenatal Medicine, Gynecological Ultrasound and Fetal Surgery, Department of Obstetrics and Gynecology, Medical Faculty and University Hospital Cologne, University of Cologne, Cologne, Germany; 10 Department of Pediatric Nephrology, Amsterdam UMC-Emma Children’s Hospital, Location University of Amsterdam, Meibergdreef 9, 1105 AZ Amsterdam, The Netherlands

**Keywords:** Lower urinary tract obstruction, Fetal medicine, Vesico-amniotic shunting, Precision medicine, Multidisciplinary management

## Abstract

**Abstract:**

Congenital lower urinary tract obstruction (cLUTO) describes a heterogeneous spectrum of congenital lower urinary tract defects with variable postnatal outcomes, ranging from high morbidity and mortality to spontaneous resolution. In the past, fetal intervention studies aimed at mitigating the disease sequelae of cLUTO have yielded inconclusive results, which contributed to the current heterogeneous antenatal management of fetuses with cLUTO across fetal surgery centers. The recent development of first-trimester diagnostics and early vesico-amniotic shunting (VAS) (i.e., < 17 weeks of gestation) in Germany to decompress the urinary tract and preserve amniotic fluid volume throughout pregnancy has added another dimension to this heterogeneity, as retrospective studies suggest a potential benefit of this procedure for overall survival as well as postnatal pulmonary and kidney function. Despite these promising results, many questions remain unanswered before early VAS can be implemented as a standard antenatal treatment for cLUTO. These questions need to be addressed by large-scale, multidisciplinary prospective studies, which are difficult to conduct for various reasons. Here, we describe the current state of the art in cLUTO management, providing a multidisciplinary perspective that includes risks and benefits of early fetal medicine approaches in the clinical management of affected newborns. Furthermore, we outline future directions to overcome challenges in optimizing our approach to improve outcomes for children with cLUTO.

**Graphical abstract:**

**Figure:**
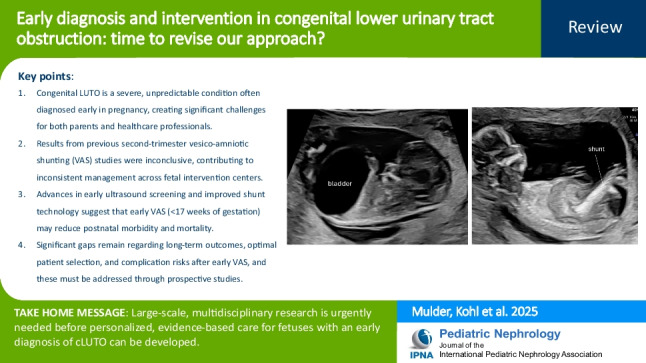
A higher resolution version of the Graphical abstract is available as Supplementary information

**Supplementary Information:**

The online version contains supplementary material available at 10.1007/s00467-025-06994-w.

## Introduction

Congenital lower urinary tract obstruction (cLUTO) refers to a heterogeneous spectrum of congenital conditions characterized by subvesical obstruction of variable severity [1]. The estimated incidence of cLUTO is 2–3 per 10,000 live births [2], with posterior urethral valves (PUV) accounting for approximately 60% of cases [3]. Other rare causes of LUTO are urethral hypoplasia/atresia/duplication, megalourethra, and prolapsing ureterocele, as well as more complex congenital defects such as Prune Belly Syndrome (PBS), Megacystis Microcolon Intestinal Hypoperistalsis Syndrome (MMHIS), and uro-ano-genital (cloacal) malformations. Unlike PUV, all of these latter phenotypes can also occur in females.

Although the exact etiology of cLUTO remains largely unknown [4–10], the clinical consequences are well documented. Oligohydramnios or anhydramnios before 20 weeks of gestation leads to aberrant canalicular lung development and pulmonary hypoplasia, making it a strong second-trimester predictor of neonatal death [11]. Several biomarkers have shown promise for predicting postnatal kidney function, but their clinical use is currently limited due to insufficient validation in prospective cohorts [12–16]. Since approximately 60% of children with cLUTO will develop chronic kidney disease (CKD) stage 3 or higher [17], expectant parents of a fetus diagnosed with cLUTO, as well as their healthcare providers, are faced with a severe diagnosis with distressing uncertainty about the prognosis.

Previous second-trimester fetal interventions to decompress the bladder and urinary tract and preserve amniotic fluid volume by vesico-amniotic shunting (VAS) have not robustly shown improvement in the postnatal prognosis of affected individuals [18, 19], contributing to the current European recommendation that VAS should be offered in selected cases [1]. Proposed indication criteria for VAS mainly encompass findings suggestive of abnormal fetal kidney function, e.g., oligo/anhydramnios, hyperechogenic kidneys, and cortical cysts [20], but have not been validated by prospective studies [20]. The recent development of early VAS (i.e., before the 17th gestational week) introduces another dimension to the desire for accurate outcome prediction in cLUTO. For example, fetuses with cLUTO often have complex lower urinary tract phenotypes, which cannot always be identified at the time of intervention, but severely impact the overall prognosis [21]. This could make the determination of who will benefit from early VAS, who will not, and at what cost problematic. Hence, we, as multidisciplinary health care professionals, are now compelled to reconsider our approach to managing fetuses with cLUTO.

In this review, we describe the current state of the art in the management of children with cLUTO, providing a balanced multidisciplinary perspective that includes potential benefits and complications of first trimester diagnosis and VAS for the postnatal outcome of affected newborns. We also highlight the associated challenges that accompany clinical decision-making regarding early VAS of children with cLUTO, which should be based on individualized risk–benefit ratios, ethical considerations, and stratified longitudinal follow-up on pulmonary, kidney, and urinary tract outcomes [20]. Accordingly, we call for international prospective initiatives aimed at improving clinical outcomes for these children.

## Antenatal management of cLUTO

### Diagnostic criteria and natural course of cLUTO

**Fig. 1:**
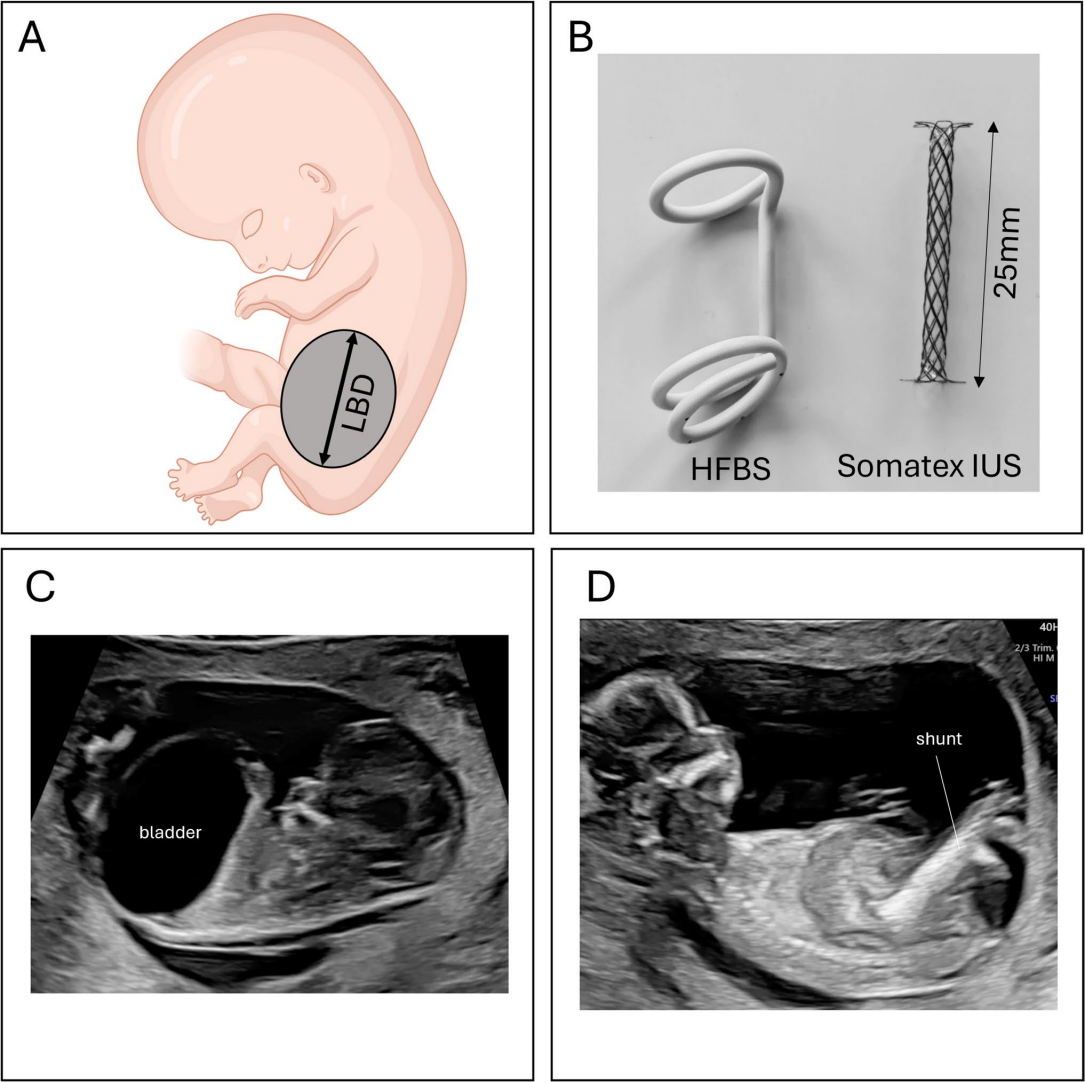
Fetal ultrasonography in congenital lower urinary tract obstruction. **A** Measurement of longitudinal bladder diameter in a first-trimester fetus with megacystis. **B** Image of two commonly used vesico-amniotic shunt systems: Harrison Fetal Bladder Stent (HFBS; left) and Somatex® intrauterine shunt (IUS; right). The shunts are deployed in the picture. **C** Ultrasound picture of a fetus with first-trimester megacystis (image courtesy of Eva C. Weber). **D** Ultrasound picture of a fetus after first-trimester vesico-amniotic shunting with the Somatex® IUS showing a completely drained bladder (image courtesy of Eva C. Weber)

Fetal megacystis, the prenatal hallmark ultrasonographic finding of LUTO, is defined as an abnormal enlargement of the urinary bladder. Up to 80% of cases of megacystis can be already detected at the first-trimester scan [22], when fetal megacystis is defined by a Longitudinal Bladder Diameter (LBD), measured in the midsagittal plane alongside the crown–rump length, of ≥ 7 mm (Fig. 1). It is important to consider that the implementation of the first-trimester scan in population screening programs still varies across countries (Table 1) [23].

**Table 1:** First trimester screening for megacystis across European countries (adapted from [23])

Country	Timing (gestational weeks)	Reimbursement
Denmark	10 to 14	Fully paid by insurance or government
Finland	13 to14	Fully paid by insurance or government
France	10 to 14	Partially covered by the national health insurance system
Germany	9 to 12 (covered) 12 to 14 (self-paid)	Partially covered by the national health insurance system, partially self-paid
Italy	10 to 14	Public sector paid by government, private sector fully self-paid, or by insurance
Netherlands	12 to 14	Fully paid by insurance or government (implementation decision pending)
Poland	11 to 14	Public sector paid by government, private sector fully self-paid, or by insurance
Spain	10 to 14	Fully paid by insurance or government
Sweden	11 to 14	Public sector paid by government, private sector fully self-paid, or by insurance
United Kingdom	10 to 12	Fully paid by insurance or government

NB. These timelines are general guidelines and may vary based on individual circumstances, healthcare provider practices, and regional policies

In the second and third trimesters, the diagnosis of megacystis has long been more subjective, and only more recently, reference charts for normal fetal bladder dimensions have been developed [24]. The suspicion of cLUTO is generally based on a persistently enlarged bladder that does not empty during the scan and appears disproportionately large relative to fetal abdominal size. Associated findings such as the keyhole sign (dilated bladder neck and posterior urethra), bilateral hydronephrosis, oligo-/anhydramnios, or bladder wall thickening support the diagnosis of LUTO.

The results of retrospective studies that have evaluated the postnatal outcome of conservatively treated cases with a first-trimester LBD > 15 mm are summarized in Table 2, demonstrating that, while spontaneous resolution may occur in isolated cases, neonatal survival rates are extremely low and pregnancy termination rates are high [25–30]. In contrast, fetuses with LBD 12–15 mm have a much more variable outcome, including spontaneous resolution [31–33]. The latter dimensions indicate a “grey zone,” where LBD appears to not be a reliable sole prognostic indicator.

**Table 2:** Overview of published cohorts reporting outcomes in conservatively treated fetuses with a first-trimester longitudinal bladder diameter > 15 mm

Reference	Cases	Cohort characteristics	Time period	Cases with 1 st trimester megacystis > 15 mm	Prenatal death	Liveborns	Neonatal survivors	Isolated 1 st trimester megacystis > 15 mm and favourable outcome?
[31]	43	Retrospective single center	2009–2020	14	12	2	2	Not available
[27]	27	Retrospective single center	2015–2023	2	2	0	0	None
[25]	145	Retrospective single center	1992–2002	31	31	0	0	None
[94]	23	Retrospective single center	2011–2016	11	11	0	0	None
[29]	5	Retrospective multi-center	2007–2015	2	0	2	2	None
[95]	98	Retrospective single center	2010–2020	22	20	2	Not available	Not available
[96]	1 (others with VAS)	Retrospective single center	2008–2012	1	0	1	0	None
[28]	2 (others with VAS)	Retrospective single center	2008–2012	2	1	1	1	1
[97]	46	Retrospective study	2003–2008	9	8	1	1	None
**Total**	**390**	**Not applicable**	**1992–2023**	**94**	**85**	**9**	**6**	**1**

This table summarizes retrospective studies assessing fetuses with an LBD > 15 mm diagnosed during the first trimester of gestation, who were not treated with vesico-amniotic shunting. Reported outcomes include prenatal death, live birth, and neonatal survival. The final column indicates whether any evidence of isolated first-trimester megacystis with LBD > 15 mm and a favorable outcome (i.e., survival without severe postnatal morbidity) was identified in the respective cohort. The data illustrate that spontaneous resolution and favorable postnatal outcomes in fetuses with isolated megacystis and a first-trimester LBD > 15 mm are exceedingly rare

*LBD* Longitudinal bladder diameter, *VAS* vesico-amniotic shunting

### Fetal interventions to improve the prognosis of cLUTO

For decades, fetal surgeons have sought to alter the natural course of severe cLUTO by introducing VAS systems with two primary aims: (1) to restore amniotic fluid to promote lung maturation and prevent pulmonary hypoplasia to reduce mortality; and (2) to preserve kidney function by relieving urinary tract pressure during nephrogenesis. The first available shunt systems include the double-pigtail Harrison Fetal Bladder Stent (HFBS; used in the second trimester and the PLUTO trial [19] (Fig. 1)), which was placed using a 13G trocar and suitable for VAS in the second trimester. Since 2014, the double-umbrella Somatex® Shunt, inserted via a 50% smaller 18G needle, enables earlier intervention (11 + weeks vs. 17 + weeks) with fewer complications than HFBS (Fig. 1) [34]. Somatex® shunt placement can be performed under ultrasound guidance as the megacystis is relatively easily accessible. The needle containing the shunt punctures the fetal bladder via the maternal abdomen, uterus, amniotic cavity, and fetal abdominal wall [34, 35]. Once the preloaded needle is positioned intravesically, the proximal umbrella is relieved using a pusher. As the needle is withdrawn, the distal end is relieved into the amniotic cavity. Adequate amniotic fluid is needed for distal positioning, which is usually still present in the first trimester. However, in cases of oligo-/anhydramnios, amnioinfusion may be necessary to enable adequate placement. Successful decompression causes immediate urinary drainage visible on ultrasound. Because the Somatex® introduction needle is blocked by the shunt, fetal urine cannot be aspirated before placing the shunt. After the shunt is deployed from the needle, which is then located in the amniotic cavity, amniotic fluid can be sampled. Worth mentioning, the newly developed Vortex shunt, that has been designed to decrease dislodgement rates and improve shunt deployment via a 7 Fr trocar, has recently been tested in a pre-clinical model of LUTO in fetal lambs and demonstrated short-term safety and functionality [36], indicating a promising first step towards the use of this device in human cLUTO.

#### Clinical outcomes after VAS

Several trials and cohort studies have assessed the safety and efficacy of VAS [19, 22, 37], with the randomized controlled PLUTO trial showing a statistically non-significant improvement in neonatal survival following second-trimester VAS [19]. However, recruitment challenges, mainly due to parental reluctance to continue the pregnancy and/or to participate in a randomized trial, led to premature termination and limited the study’s power [19, 38]. Meta-analyses of second-trimester VAS studies reported survival rates of 30–60%, with poor long-term renal outcomes [37, 98], which may be an indication that the intervention was performed at too advanced a stage to be beneficial for many cases. Moreover, data from animal studies have demonstrated an association between the severity of postnatal kidney dysfunction and the duration of urinary obstruction, with early decompression preserving kidney function [39–41].

Based on these findings, some centers have explored earlier VAS using the Somatex® shunt [34, 42–44]. Retrospective analyses from German centers report better survival and kidney outcomes in fetuses with cLUTO diagnosed and treated with VAS before 17 weeks compared to those diagnosed and treated later than 17 weeks [34, 44, 45]. Strizek and co-workers compared the perinatal outcomes of VAS before the 17th gestational week with the HFBS vs. Somatex® shunt in 57 fetuses with isolated cLUTO and identified normal kidney function in 7/8 (88%) of children in the HFBS group (median age at last follow-up 7.3 years) and 17/25 (68%) in the Somatex® group (median age at last follow-up 2.9 years; *P* = 0.39) [34]. Two (25%) children in the HFBS group had kidney failure compared to 6 (24%) children in the Somatex® group. Kohl et al. performed VAS using the Somatex® shunt or a pigtail catheter (Cook medical®) and calculated an odds ratio of 7.9 (95% confidence interval 2.0–30.8) for normal kidney function at last follow-up when VAS was performed before vs. after the 17th gestational week (*P* = 0.003) [44]. An observational study of 9 fetuses that were treated with VAS between gestational weeks 14 + 6 and 27 + 6 reported survival of 6 fetuses at follow-up (median age 1.8 years), of whom 1 child was treated before the 17th gestational week and had moderately impaired kidney function [45]. Overall, kidney failure rates after early VAS range from 0–24% [34, 42, 43, 45]. Gottschalk et al. did not find differences in restricted kidney function after birth between groups treated < 14 (0%), 14– ≤ 17 (14%), and > 17 (38%) weeks of gestation, but a numerical trend favoring earlier intervention was observed (*P* = 0.089) [43]. Preliminary data from the prospective IUS1st trial (DRKS00017779), studying first-trimester megacystis with LBD > 15 mm treated at a median of 13.8 gestational weeks, suggest improved postnatal kidney outcomes despite persistent urological issues (data unpublished).

As all of the current studies describe retrospective case series with relatively short follow-up periods, differences in, e.g., procedure and timing of the intervention as well as termination of pregnancy rates, larger-scale prospective studies are needed to confirm these results. However, randomized trials of fetal interventions face extreme challenges due to selection bias, high dropout rates, lack of adequate controls, treatment cross-over, and the need for long-term follow-up (i.e., multiple decades in the case of cLUTO) [19]. Given these difficulties, the current introduction of early VAS highlights the need to better define which fetuses would benefit most from a fetal intervention [20]. Of note, the Somatex® shunt has been currently used in a limited number of countries across Europe and Asia [46–48]. Overcoming this limited availability of fetal shunting systems will be essential to answer this pivotal question in the treatment of fetuses with cLUTO.

#### Risks and complications of early VAS

Like any intrauterine intervention, VAS carries risks such as fetal loss, premature rupture of membranes, amniotic fluid leakage, preterm labor and birth, although hazard risks have not yet been substantiated for early VAS. VAS-specific complications include shunt dislocation and occlusion, which may require re-shunting. The dislocation rate varies by shunt type, with the Somatex® Shunt showing lower rates than the Harrison Fetal Bladder Stent (27% vs. 63%) [34]. Rare complications include fetal bleeding, skin defects from mechanical abrasion, intestinal injury, and iatrogenic fetal abdominal wall defects at the insertion site [35, 49]. Premature rupture of membranes has been described in 8% of pregnancies, while others have identified higher rates (30%) of preterm delivery before the 32nd gestational week [43, 49].

Particularly for first-trimester VAS, selection of an optimal shunt insertion site caudal of the umbilicus is important. As the fetus grows and the bladder remains decompressed, the bladder and abdominal wall puncture sites diverge over time. Once the distance between these sites exceeds the shunt length, the amniotic umbrella may slip into the peritoneal cavity, leading to urinary ascites. This complication typically occurs > 20 weeks of gestation and may require additional abdomino-amniotic shunt placement.

Postnatal complications of VAS with a Somatex® shunt have been recently described by several retrospective case series [34, 35, 49]. Messling et al. reported that postnatal surgical removal of the shunt was indicated in 13/23 (56%) fetuses who underwent early VAS [49]. For five neonates, this procedure was described as “complex.” Kohaut et al. identified similar proportions of postnatal shunt removal (10/17 (58%) neonates with cLUTO after early VAS) [35]. Other shunt-related complications include dislocation (15/23, 65%), dysfunction (3/23, 13%), abdominal wall defects (3/23, 3%), intestinal adhesions (4/23, 17%), bladder adhesions (1, 4%), urinary ascites (2/23, 9%), and aplasia cutis (1/23, 4%) [49]. Although the published complication rates of early VAS appear substantial, these must be weighed against the outcome of conservatively treated children with severe LUTO and the improved kidney function compared to later VAS. Nevertheless, until larger prospective cohorts further uncover the complication rates of early VAS, individualized clinical decision-making is warranted for fetuses with LBD > 15 mm.

#### Alternative prenatal management strategies

In 2024, an international Delphi consensus panel reaffirmed VAS as the primary intervention for fetuses with megacystis [30]. Some centers have explored a curative approach, targeting PUV as the commonest cause of cLUTO in male fetuses. While postnatal PUV is treated by cystoscopic incision or resection, prenatal fetoscopic cystoscopy with antegrade laser fulguration has largely been abandoned due to the high severe complication rate and technical challenges [50].

Serial vesicocentesis, performed with a thin needle, was also considered but abandoned as it only temporarily reduced urinary pressure, while it may lead to urinary ascites and does not prevent pulmonary hypoplasia. Other risks of this procedure include premature rupture of membranes, preterm labor, and infection.

Serial amniotic fluid infusion aims to prevent pulmonary hypoplasia by maintaining amniotic fluid volume but does not relieve urinary pressure and carries high prenatal complication risks. The Renal Anhydramnios Fetal Therapy Trial showed that serial amnioinfusions reduced lethal pulmonary hypoplasia in fetuses with bilateral kidney agenesis, but led to a high rate of preterm delivery and mortality unrelated to lung function [51].

Finally, healthcare providers should always consider and discuss with the expectant parents the option of foregoing antenatal or postnatal intervention in favor of providing optimal comfort care after birth.

## Postnatal clinical management of children with cLUTO

**Fig. 2:**
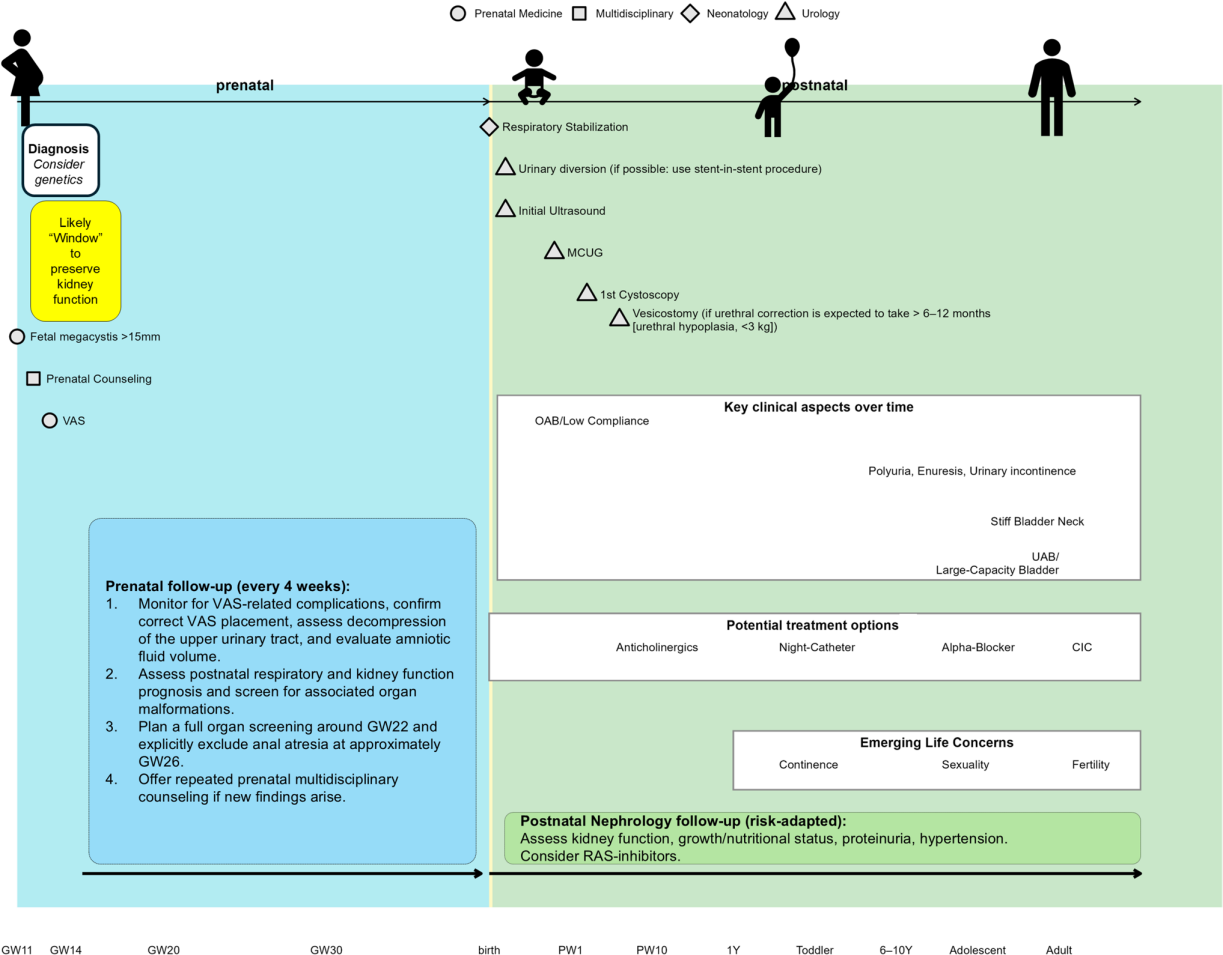
Multidisciplinary management of congenital lower urinary tract obstruction following early vesico-amniotic shunting. For details about the antenatal and postnatal follow-up, see the respective sections in the manuscript. The recommended prenatal counseling team should at least include medical specialists trained in obstetrics, fetal surgery, medical ethics, clinical genetics, neonatology, pediatric nephrology, and pediatric urology. CIC = clean intermittent catheterization; FU = follow-up; GW = gestational week; MCUG = micturating cystourethrogram; OAB = overactive bladder; RAS = renin–angiotensin–aldosterone system; UAB = underactive bladder; VAS = vesico-amniotic shunting

Management of infants with cLUTO requires specialized and multidisciplinary care due to the broad spectrum of pulmonary and kidney dysfunction, which depends on the timing, duration, and severity. Over recent decades, neonatologists, pediatric nephrologists, and urologists have integrated the complex management of patients with cLUTO into their practice [1]. An example of the current antenatal and postnatal management for children with cLUTO treated with early VAS is summarized in Fig. 2.

### The neonatological management of cLUTO

While neonatal care for very preterm infants has dramatically improved over the past years, this does not particularly concern term or preterm infants with pulmonary hypoplasia due to cLUTO.

As of now, no systematic study has been published that evaluated the optimal pulmonary management of newborns born with cLUTO in the first days or weeks of life [52]. Hence, current neonatal management recommendations are extrapolated from experience with infants having pulmonary hypoplasia due to kidney dysfunction by other causes. Here, the initial postnatal care depends on expected pulmonary function, which is mainly assessed based on the amniotic fluid volume. Adequate amniotic fluid until at least 22 weeks’ gestation allows for sufficient lung maturation to support gas exchange at birth [53]. In fetuses with normal amniotic fluid, vaginal delivery and regular postnatal bonding may still be pursued, even with a VAS in place. Pulmonary management in the case of pulmonary hypoplasia as a consequence of oligo-/anhydramnios should be as minimally invasive as possible to avoid early pulmonary shear trauma or pneumothoraces but may include high-frequency oscillation (with or without the need for nitric oxide and milrinone) and extracorporeal membrane oxygenation as a bridge treatment for severe postnatal pulmonary failure. Given this high interindividual variability of postnatal pulmonary function, we strongly advocate for the prenatal involvement of dedicated neonatologists in the clinical management of every child with cLUTO (Fig. 2).

In addition to pulmonary management, achieving optimal nutritional status and growth after birth is essential to ensure neurocognitive development and may be complicated in children with cLUTO due to acid–base disorders, electrolyte imbalances, and chronic dehydration associated with decreased postnatal kidney function. As a consequence, many children with cLUTO require gastric tube feeding and caloric supplementation after birth [54].

With regard to early VAS, it remains to be determined whether this will alter specific aspects of the abovementioned neonatal care for children with cLUTO, making this truly different from non-shunting or late VAS. According to our own clinical experience, this change has been limited to the removal of the shunt system and preventing urinary outflow obstruction, as will be described below.

### The nephrological management of cLUTO

Postnatal nephrological management of children with cLUTO ranges from supportive treatment (i.e., increased fluid intake, electrolyte supplementation) to kidney replacement therapy, e.g., peritoneal dialysis. Severe volume and electrolyte imbalances—such as hyponatremia, hyperkalemia, and acidosis, commonly associated with impaired kidney function in non-shunted or late-shunted cLUTO cases [55, 56]—are less often observed in neonates with early VAS. However, the long-term effects of early VAS placement on CKD remain unclear due to limited long-term follow-up data [57], and it is reasonable to assume that many affected children still have a more or less reduced nephron endowment, increasing their risk for CKD-related complications later in life. Still, this would mean a significant decrease in the complexity of nephrological management compared to a child born with kidney failure resulting from cLUTO (Fig. 2). For children treated with early VAS who have normal kidney function after birth, general management recommendations established for both patients with cLUTO and patients with reduced nephron number are likely applicable [1, 58]. Preventing obesity and treatment with renin–angiotensin–aldosterone system inhibition in cases of hypertension and/or proteinuria may be indicated to slow down glomerular filtration rate deterioration [59]. Children with cLUTO are also at higher risk for kidney injury from urinary tract infections and persistent lower urinary tract dysfunction, which can worsen long-term kidney prognosis. Importantly, children initially showing well-preserved kidney function face risks of loss to follow-up and, consequently, delayed detection of these modifiable CKD progression factors.

### The urological management of fetal cLUTO

All newborns with cLUTO should undergo postnatal bladder drainage following initial stabilization. In VAS-treated infants, shunt removal is generally non-urgent and should not take precedence over necessary pulmonary management or early bonding [60]. If a Somatex® shunt is still functional after birth, a “stent-in-stent” procedure (e.g., using a 5 French tube) can be employed to maintain bladder drainage without an additional invasive procedure. If the shunt is not functional or inaccessible and the bladder is distended, placement of a suprapubic catheter is advised, particularly if transurethral catheterization is unsuccessful [60]. If concomitant anal atresia is present, shunt removal and suprapubic catheter placement may be performed in conjunction with first-day colostomy.

Once bladder drainage is secured and the infant is clinically stable, a micturating cystourethrogram is performed to determine the underlying cause and guide further management [61], e.g., early endoscopic valve ablation in at term boys with PUV or (temporary) urinary diversion in premature infants (< 3.0 kg) or children with urethral hypoplasia or atresia [62–64]. Early VAS may impair urethral development due to lack of urinary flow in the first trimester, which is believed to maintain lumen patency and elasticity [21, 65]. This might contribute to the high incidence of urethral hypoplasia reported in patients with early VAS [21]. Differentiating between PUV, urethral atresia, and congenital urethral stenosis or hypoplasia as the underlying cause of bladder outlet obstruction can be challenging in such cases. Moreover, early attempts at urethral dilation may induce iatrogenic stenosis, which could later be misclassified as congenital urethral hypoplasia. Therefore, early diversion by suprapubic catheter placement or vesicostomy is advised in such cases until definitive treatment is feasible. Vesicostomy offers easier home management and avoids repeated anesthesia compared to a suprapubic catheter, though some argue it may alter bladder function [66–68].

Data on achieving daytime and nighttime urinary continence are conflicting and missing for patients with cLUTO treated by VAS. Some reported comparable results to their peers [69], while others show delays, particularly in those with long-term diversions or urethral reconstruction [70]. In addition, a subset of patients may require appendico-vesicostomy for bladder emptying in the long-term [71]. Circumcision is often beneficial for UTI prevention [72], but should be deferred in patients where the foreskin may be needed for urethral reconstruction [73].

The latest EAU pediatric guidelines recommend lifelong monitoring of bladder and kidney function in patients with cLUTO [74] (Fig. 2). Overnight bladder drainage via catheter has shown benefits in improving kidney function-related outcomes, continence, hydronephrosis, and reflux [75]. Anticholinergics and alpha-blockers are effective for managing detrusor overactivity, low bladder compliance, and functional outflow obstruction [76]. A recent meta-analysis found no significant differences in long-term kidney or bladder outcomes between primary valve ablation and urinary diversion, although data for patients with early VAS are lacking [77]. Extended follow-up data will be crucial to determine whether patients with early VAS behave similarly to non-shunted patients with subsequent long-term diversions. Sexual function and fertility outcomes in patients with PUV remain poorly studied, with limited, non-representative data on semen parameters [78]. Preliminary reports suggest that sexual function is not significantly impaired [79–81]. In line with the nephrological management, the urological follow-up of children who have undergone early VAS is recommended to be lifelong.

## Ethical aspects of fetal intervention in cLUTO

Antenatal treatment of cLUTO has been debated since it became technically feasible. The ethical dilemma surrounding fetal interventions in cLUTO centers on balancing potential benefits and harms, respecting parental autonomy and informed consent, and considering the duties of healthcare professionals toward both the pregnant woman and the fetus. Noble et al. suggest that fetal therapy may be justifiable when: (1) there is a high likelihood that the fetus will experience significant and irreversible harm without intervention; (2) the intervention is proven effective; (3) the risk to the pregnant woman’s health and well-being is minimal; and (4) the pregnant woman is capable of providing valid informed consent for the intervention [82]. Broader ethical and societal considerations, including quality of life and the acceptability of pregnancy termination, also shape this discussion.

Prenatal therapy in cLUTO is not routine and must be tailored to each individual case, taking into account the severity of cLUTO, the likelihood of a poor prognosis without intervention, the potential benefits of the procedure, minimal and acceptable risk to the expectant mother, the ability to obtain fully informed and voluntary consent from the parents, and support for the intervention by the multidisciplinary team. This individualized approach is reflected in the recommendations of ERKNet and NICE (https://www.nice.org.uk/guidance/ipg202/resources/fetal-vesicoamniotic-shunt-for-lower-urinary-tract-outflow-obstruction-pdf-1899865151221957) to consider VAS in carefully selected cLUTO cases [1]. Still, differences in actions after the PLUTO trial results illustrate the complexity of this selection. Although the trial did not demonstrate clear benefits of VAS placement over non-intervention [19], it led to the abandonment of the procedure in The Netherlands, while Germany continued research supporting earlier intervention enabled by advances in VAS systems. This has resulted in a growing—although retrospective and uncontrolled—body of evidence indicating relatively high neonatal and kidney function survival in high-risk cases (e.g., LBD > 15 mm) with minimal maternal risk [34, 44, 45].

Clinicians now face a dilemma: to proceed without definitive trial data relying on high-risk cohort observations or to delay intervention until better evidence is available, despite untreated cLUTO frequently resulting in a severe prognosis. For some parents, survival justifies fetal intervention, while others may opt for pregnancy termination based on personal perceptions of their child’s quality of life, even when kidney replacement therapy after birth might be avoidable. For example, early VAS is currently performed more reluctantly in females with complex cloacal malformations that are difficult to repair [43]. Identification of such complex anomalies after early VAS independently worsens the prognosis and is often undetectable in the first trimester [21]. Therefore, it is crucial that parents are counseled about the possibility of pregnancy termination or postnatal palliative care after VAS. Decisions become even more complex in milder cases (e.g., LBD 12–15 mm), where the benefits of intervention are less clear and spontaneous resolution is more likely, or when the diagnosis is made after the first trimester. We therefore strongly recommend including medical ethicists as members of the multidisciplinary team that participates in clinical decision-making regarding early VAS in fetuses with cLUTO (Fig. 2).

## Future perspectives to advance the treatment of cLUTO

Conducting randomized controlled trials (RCTs) in cLUTO is hindered by the rarity of the condition, expectant parents who do not consent for randomization, and frequent pregnancy terminations, while long-term follow-up is necessary to assess kidney, urinary tract, and pulmonary outcomes. Despite very similar barriers, RCTs and prospective studies with antenatal interventions in other conditions—such as twin-to-twin transfusion syndrome, congenital diaphragmatic hernia, and spina bifida—have been conducted and led to changes in clinical management [38, 83, 84]. However, the natural course of these conditions as well as the trial design and circumstances under which they were conducted were not one-to-one comparable to the cLUTO perspective.

Van Mieghem et al. cautioned that long-standing uncertainty about the indications and efficacy of a fetal intervention may drive practitioners to form strong opinions based on personal experience—a concern increasingly relevant in the light of early VAS [38]. Therefore, we urgently advocate for a well-designed large-scale prospective observational study with matched controls (e.g., from centers that currently do not perform fetal interventions) and long-term nephro-urological follow-up (ideally into adulthood) as the next best option to optimize our approach for cLUTO. We propose that these studies will be carried out in specialized fetal intervention centers that are integrated within larger research consortiums. Conducting research in this way would most effectively address the central challenge of identifying fetuses who genuinely benefit from early VAS, while carefully weighing the associated risks and complications.

Better understanding of the molecular basis for cLUTO could aid in selecting optimal candidates for VAS. While a few monogenic causes have been described [4, 8], insights into the exact etiology remain limited for most cLUTO cases [85] and impede genetic counseling of expectant parents. Genetic testing (e.g., for MMIHS) may influence postnatal treatment and should be considered, especially when extrarenal malformations are present in the fetus. Rapid genomic testing via amniocentesis (e.g., for trisomies 21/18/13 or 22q11.2 microdeletions) can provide critical prognostic insight. Emerging research suggests cLUTO may represent a continuum of phenotypes related to diverse genetic mechanisms—monogenic, structural, or multifactorial [9, 86–89]. Despite that the identification of pathogenic variants by research will likely take decades, this may eventually enhance prenatal counseling and treatment decision-making.

To predict postnatal kidney outcomes, fetal urine peptides, cytokines, and metabolomic biomarkers (identified in the second trimester) show promise [16, 90, 91]. Applying and validating these approaches before 17 weeks could be transformative for early VAS decision-making in cLUTO. The Somatex® shunt prevents fetal urine sampling during intervention, but amniotic fluid can be aspirated. Recently, amniotic fluid peptides, but not cytokines, were demonstrated to accurately predict postnatal kidney function in 12 children with CAKUT phenotypes (including PUV) [15, 16], indicating the need for future validation studies to establish biomarker profiles for postnatal kidney function when early VAS is used. Establishing international and multicenter data- and biobank initiatives is the first step to come towards such objective postnatal outcome prediction in cLUTO.

Hence, we strongly support the expansion of international registries that integrate prenatal and postnatal data, including long-term follow-up and antenatal losses, as exemplified by the IUS1st trial (DRKS00017779). Although scientific networks such as ERKNet, eUROGEN, and ESPN-ERA-EDTA are well positioned to lead these efforts, current registries often fail to capture terminations or intrauterine deaths as well as multidisciplinary data end-points.

To address this gap, we highlight the CaRE for LUTO Registry (www.careforluto.de), which was specifically established to combine prenatal data with multidisciplinary postnatal long-term follow-up. We also support the RESUME Registry [92], a global retrospective registry focused on fetuses with cLUTO treated antenatally. We encourage the continued development and expansion of such large-scale collaborations to improve the clinical management of children with cLUTO.

## Conclusions

In conclusion, cLUTO is a potentially severe and unpredictable condition, imposing an extreme emotional burden on expectant parents and complex challenges for healthcare providers. Previous attempts to decompress the urinary tract using VAS in the second trimester have yielded inconsistent results, contributing to varied clinical practices across fetal intervention centers. However, with the advent of routine first-trimester ultrasound screening and advances in shunt technology, recent retrospective data suggest that early VAS placement may reduce postnatal morbidity and mortality.

Despite these advances, critical uncertainties remain regarding long-term outcomes, optimal patient selection, and the risks of complications associated with early intervention. To address these gaps and improve clinical management, large-scale, multidisciplinary studies must be urgently initiated. These should aim to develop personalized treatment strategies that optimize care for children with cLUTO and provide meaningful support for their families.

## Key summary points

1. Congenital LUTO is a severe, unpredictable condition often diagnosed early in pregnancy, creating significant challenges for both parents and healthcare professionals.
2. Results from previous second-trimester VAS studies were inconclusive, contributing to inconsistent management across fetal intervention centers.
3. Advances in early ultrasound screening and improved shunt technology suggest that early VAS (< 17 weeks of gestation) may reduce postnatal morbidity and mortality.
4. Significant gaps remain regarding long-term outcomes, optimal patient selection, and complication risks after early VAS, and these must be addressed through prospective studies.
5. Large-scale, multidisciplinary research is urgently needed before personalized, evidence-based care for fetuses with an early diagnosis of cLUTO can be developed.

## Supplementary Information

Below is the link to the electronic supplementary material.

Graphical abstract (PPTX 1011 KB)
