# Supplementary material for: Early diagnosis and intervention in congenital lower urinary tract obstruction: time to revise our approach?
Source: Pediatr Nephrol. 2025 Nov 13;41(7):1937–50. doi: 10.1007/s00467-025-06994-w (PMC13197321; doi:10.1007/s00467-025-06994-w)
Supplement: Supplementary file 1 — Graphical abstract (PPTX 1011 KB) [file 467_2025_6994_MOESM1_ESM.pptx]

## Slide 1
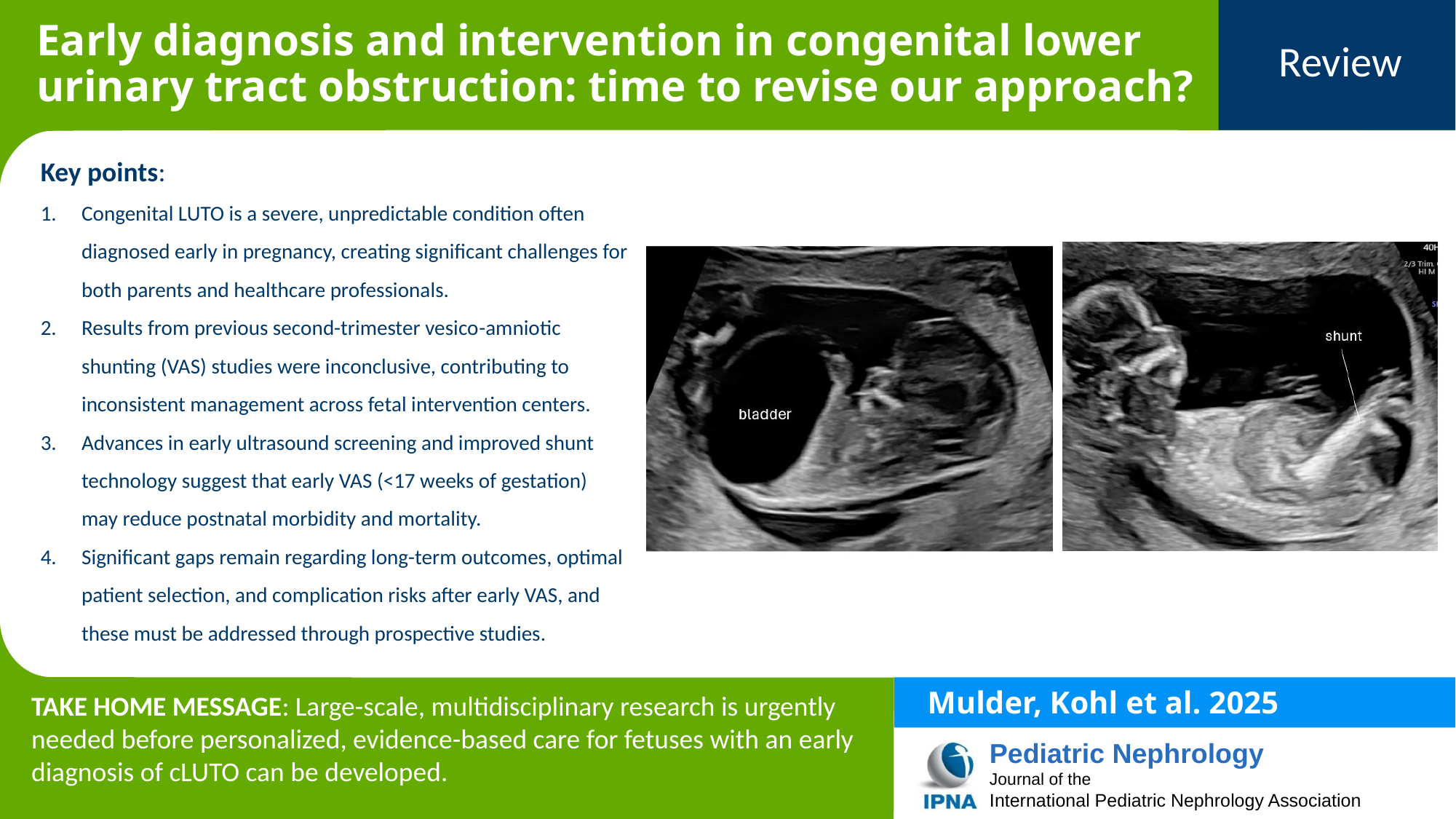

Early diagnosis and intervention in congenital lower urinary tract obstruction: time to revise our approach?
Key points:
Congenital LUTO is a severe, unpredictable condition often diagnosed early in pregnancy, creating significant challenges for both parents and healthcare professionals.
Results from previous second-trimester vesico-amniotic shunting (VAS) studies were inconclusive, contributing to inconsistent management across fetal intervention centers.
Advances in early ultrasound screening and improved shunt technology suggest that early VAS (<17 weeks of gestation) may reduce postnatal morbidity and mortality.
Significant gaps remain regarding long-term outcomes, optimal patient selection, and complication risks after early VAS, and these must be addressed through prospective studies.
Mulder, Kohl et al. 2025
TAKE HOME MESSAGE: Large-scale, multidisciplinary research is urgently needed before personalized, evidence-based care for fetuses with an early diagnosis of cLUTO can be developed.
